# Middle Ear Active Implant Indications, Comparative Audiometric Results from Different Approaches, and Coupling with the Vibrant Soundbridge^®^: A Single Center Experience over More Than 20 Years

**DOI:** 10.3390/audiolres14040061

**Published:** 2024-08-21

**Authors:** Joan Lorente-Piera, Raquel Manrique-Huarte, Janaina P. Lima, Diego Calavia, Manuel Manrique

**Affiliations:** Department of Otorhinolaryngology, Clínica Universidad de Navarra, 31000 Pamplona, Spain; rmanrique@unav.es (R.M.-H.); jpatriciode@unav.es (J.P.L.); dcalaviag@unav.es (D.C.); mmanrqeu@unav.es (M.M.)

**Keywords:** middle ear active implant, Vibrant Soundbridge, hearing loss, cholesteatoma, hearing implants

## Abstract

Background: Middle ear active implants, such as the Vibrant Soundbridge (VSB), offer an alternative to reconstructive surgery and other implantable hearing aid systems for patients with conductive, mixed, or sensorineural hearing loss. The primary objective of this work is to describe the auditory results obtained with VSB in our patient cohort, measuring the auditory gain in terms of average tonal thresholds and spoken word discrimination at 65 dB. Secondly, auditory gain differences between different types of hearing loss, coupling to the ossicular chain compared to round and oval windows, and the impact of open versus more conservative surgical approaches, were analyzed. Methods: A cross-sectional observational study, with retrospective data collection, was conducted at a tertiary care center. Clinical and audiometric data pre- and post-implantation were included, from patients who underwent VSB device placement surgery between 2001 and 2024. Results: 55 patients with an average age of 62.58 ± 17.83 years and a slight preference in terms of the female gender (52.72%) were included in the study. The average gain in the PTA for all types of hearing loss was 41.56 ± 22.63 dB, while for sensorineural hearing loss (SNHL) the gain was 31.04 ± 8.80 dB. For mixed-conductive hearing loss (C-MHL) a gain of 42.96 ± 17.70 was achieved, notably, in terms of absolute values, at frequencies of 4000 and 6000 Hz, with gains reaching 49.25 ± 20.26 dB at 4 K and 51.16 ± 17.48 dB at 6 K. In terms of spoken word discrimination, for all types of hearing loss, an improvement of 75.20 ± 10.11% was achieved. However, patients with C-MHL exhibited an approximately 13% higher gain compared to those with SNHL (69.32 ± 24.58% vs. 57.79 ± 15.28%). No significant differences in auditory gain were found between open and closed surgical techniques, nor in the proportion of adverse effects, when comparing one technique with the other. Conclusions: The VSB is effective in improving hearing in patients with mixed, conductive, and sensorineural hearing loss, with significant gains at high frequencies, especially through the round window membrane approach. The choice of surgical technique should consider the patient’s anatomical characteristics and specific needs in order to optimize auditory outcomes and minimize postoperative complications.

## 1. Introduction

Active middle ear implants (AMEIs) are hearing systems designed to transmit mechanical energy to a vibratory component of the ossicular chain or directly to the fluids in the inner ear at the round window (RW) or oval window (OW), unlike the mechanism of action of conventional hearing aids [1]. These devices offer an alternative to reconstructive surgery and other implantable hearing aid systems, such as bone conduction implants or hearing aids. AMEIs are especially recommended for individuals who cannot use traditional solutions or whose hearing loss is beyond the range of other hearing systems. This may be due to medical contraindications, such as an external ear condition that contraindicates occluding the auditory canal with hearing aids, insufficient performance of these devices as measured by speech audiometry, or surpassing the indications for bone conduction with bone conduction implants.

Subjectively, patients report better sound quality, improved localization, and notably better speech intelligibility when using AMEIs compared to other devices, such as hearing aids [2]. However, they are costly and require surgery under general anesthesia for both installation and replacement, or for the repair of potential implant failures.

AMEIs like the Vibrant Soundbridge (VSB) have emerged as a viable alternative for patients with sensorineural, mixed, or conductive hearing loss. The VSB system is surgically implanted under the skin and connected to a vibratory component (the floating mass transducer, or FMT). In terms of indications, the original recommendation for sensorineural hearing loss involves coupling the VSB to the incus, on either the long process or the body of the incus. This is particularly advised for individuals with moderate to severe high-frequency hearing loss and speech discrimination of ≥50% at 65 dB, who are either dissatisfied with their hearing aids or experience limitations in their use, such as recurrent external otitis [3,4].

Conversely, coupling the VSB to the round window is indicated for patients with conductive or mixed hearing loss. This approach is particularly recommended for cases of chronic otitis media, cholesteatomatous mastoidectomy (using either open or closed techniques), or unsatisfactory otosclerosis surgery. Special caution is advised in these instances due to the potential lack of movement in the windows, which is necessary for intracochlear fluid vibration [5,6,7]. To optimize vibration in these situations, a manufacturer-provided coupler can be used, and fascia or cartilage can be placed between the FMT and the round window. Additionally, the VSB has been used in the round window for patients with malformations, whether it be external auditory canal atresia or ossicular chain malformations. The expected auditory gain with a FMT in the round window varies between 25 dB and 60 dB, while placement in the oval window can achieve 30 to 35 dB [2]. However, the greatest functional gain with AMEIs has been demonstrated with stapes coupling, achieving up to 65 dB.

Finally, it is important to note that the performance of the VSB system can vary based on individual anatomical differences, either intrinsic or due to surgical procedures to treat the underlying pathology, as well as the efficiency of the FMT coupling [8]. The evaluation of the need to use a coupler or coupling system depends on the surgeon’s judgment, potentially introducing variability in surgical and auditory outcomes [9].

The primary objective of this study is to describe the auditory outcomes achieved with the VSB in our patient cohort, specifically measuring the auditory gain in pure tone thresholds at speech frequencies. Secondarily, we aim to confirm intergroup differences in the auditory gain among patients with different types of hearing loss (sensorineural vs. conductive–mixed hearing loss), comparing ossicular chain coupling to the RW and OW anchoring. Additionally, we seek to determine whether the placement of this middle ear active implant in a cavity treated with an open mastoidectomy technique versus a more conservative approach influences the audiometric outcomes.

## 2. Materials and Methods

### 2.1. Study Design

A cross-sectional observational study, with retrospective data collection, was conducted at a tertiary care center. This study was designed and carried out in accordance with the ethical guidelines in the Declaration of Helsinki of 1975. Written informed consent was obtained from all study participants.

### 2.2. Inclusion Criteria

**Figure 1:**
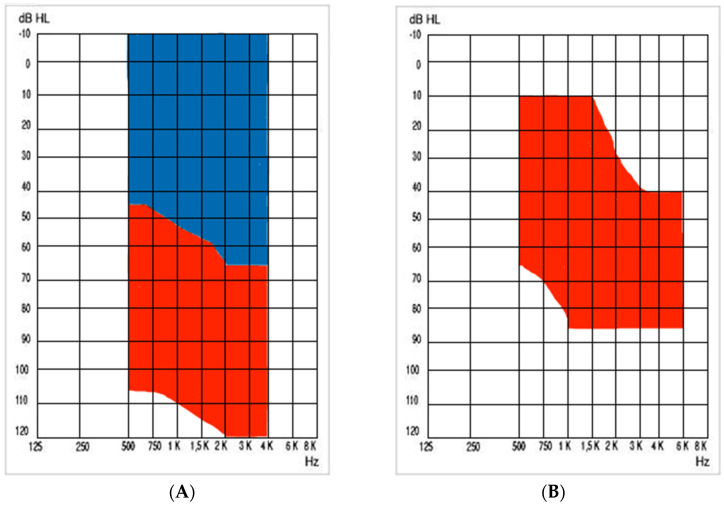
(**A**) shows the Vibrant Soundbridge indication for conductive or mixed hearing loss: the bone conduction threshold must fall within the blue shaded area on the chart, while the air conduction threshold should be within the red shaded area. (**B**) shows the indication for sensorineural hearing loss: the air conduction threshold must be within the red shaded area on the chart. In both cases, at least 50% speech understanding is required, using headphones, during an open-set word test. Courtesy of VSB MED-EL Co.

Pre- and post-implantation data were collected from subjects who underwent surgery for the placement of a Vibrant Soundbridge VORP503 device (MED-EL, Innsbruck, Austria), between 2001 and 2024. The audiological indications for mixed–conductive hearing loss, as well as sensorineural hearing loss, are represented in Figure 1.

### 2.3. Surgical Procedure

In our study, two primary approaches were used for the placement of the FMT: either coupling to the ossicular chain (incus or stapes) or through round and oval windows. Generally, the most employed procedure is the standard approach through a mastoidectomy with posterior tympanotomy, followed by anchoring the transducer to one of the previously mentioned structures. Depending on the patient, stabilization is achieved using a coupler, along with the placement of fascia or cartilage interposed between the FMT and the ossicle, or the round or oval window.

**Figure 2:**
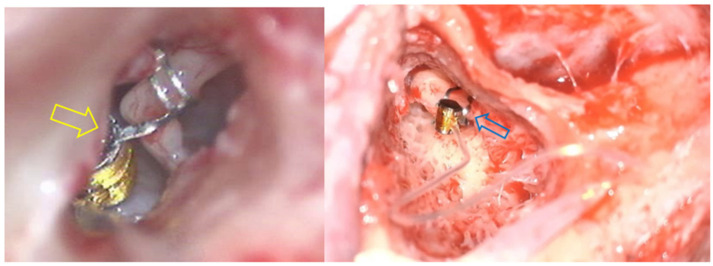
Surgery for the placement of the VSB using two different approaches. Firstly, in the left image, the yellow arrow shows the FMT anchored on the long process of the incus through a posterior timpanotomy. In the right image, the blue arrow shows the FMT anchored also on the long process of the incus, but in this case, through an extended atticotomy.

However, it is important to note that, regarding the coupling at the incus, although classically the FMT is anchored to the long process of this ossicle, in some cases, the FMT is placed on the short process of the incus through an atticotomy. Examples of different approaches to get into the incus are shown in Figure 2 [2].

Additionally, regarding cholesteatoma resection techniques, two options were considered, namely the closed technique (canal wall up) or two types of open techniques: the classic or canal wall down, with or without closure of the external auditory canal, or in some cases, an alternative, such as on-demand techniques. These were used in a considerable number of patients in our cohort, specifically attic exposure–antrum exclusion (AE-AE), a technique that completely exposes the attic by drilling the superior wall of the external auditory canal and which excludes the antrum and mastoid by closing the aditus with cartilage grafts (Manrique-Huarte et al. [10]).

### 2.4. Follow-Up and Audiometric Studies

Postoperative follow-up of the implant includes medical appointments at one week, four weeks (implant activation), three months, six months, and one year. Additionally, continuous monitoring of the otological cause is maintained. Our study encompasses the medical history and audiometric analysis at the pre-implantation stage and compares it with one-year post-implant. It also studies the surgical details and potential complications.

The key variables for comparing auditory performance with the AMEI include pre- and post-implant average tonal thresholds (PTA) and the gain difference measured in decibels at 500, 1000, 2000, 4000, and 6000 Hz, with variables measured in terms of tonal audiometry. The decision to measure different frequencies, especially high frequencies, was due to the extensive knowledge that this type of implant provides an effective level of amplification, particularly at higher frequencies [3,4]. On the other hand, through speech recognition, the gain achieved with the VSB in terms of the percentage of discrimination at 65 dB was measured, both prior to implant placement and post-implant using free-field audiometry.

### 2.5. Statistical Study

Quantitative demographic data were presented using the mean, range, and standard deviation. The normality of the data distribution was assessed using the Shapiro–Wilk test and parametric methods were used.

To compare the gains in the PTA and the verbal discrimination between different groups, *t*-tests were used. The *t*-tests conducted were paired comparison tests, as they compared the differences in speech discrimination measures for each patient at two different times: before and after the placement of the VSB. The groups were categorized based on the surgical technique used (canal wall down with AE-AE vs. canal wall up) and the type of hearing loss (SNHL vs. C-MHL) in the group with ossicular chain coupling. In the case of the overall results and the coupling at the round and oval windows, no statistical comparisons were made due to the large differences in the sample sizes between them, making it difficult to demonstrate statistical significance. Finally, to determine which type of surgical approach was associated with a higher proportion of adverse effects, Fisher’s exact test was conducted.

For the statistical analysis, a significance level of *p* < 0.05 was considered. All analyses were performed using RStudio 1.4.1106 software, ensuring the accuracy and reproducibility of the results.

## 3. Results

A total of 55 patients underwent Vibrant Soundbridge (VSB) implantation between 2001 and 2024. Of these subjects, 52.72% were women (n = 29), while the remaining 26 patients (47.28%) were men. All patients (100%) were regular users of the implant from the time of placement until the end of the follow-up. A summary of the demographic data is presented in Table 1.

### 3.1. Etiologies and Overall Results

**Figure 3:**
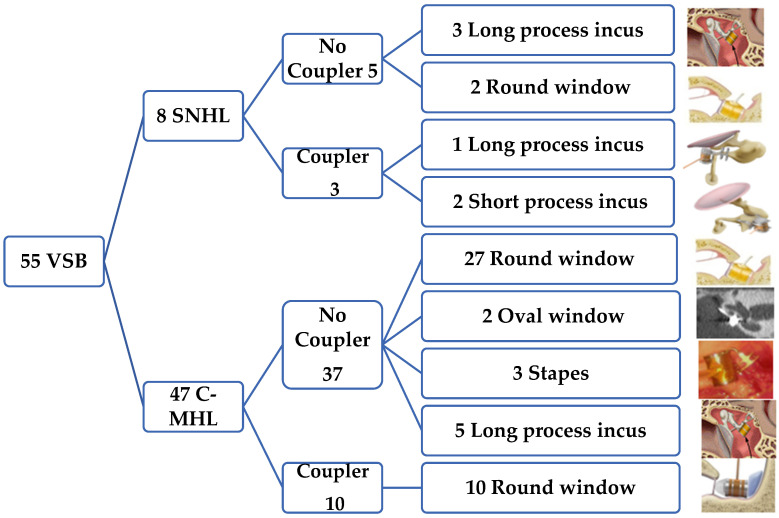
Summary of the sample distribution based on the type of hearing loss, the use of a coupler, and the FMT coupling location. The right column shows the situation both intraoperatively and in imaging tests, as well as representations that exemplify the versatility and different positions the implant can occupy, with or without a coupler, in the middle ear.

Regarding the etiologies of implantation, we found that, as seen in Figure 3, the most frequent audiometric pattern was mixed or conductive hearing loss in a total of 47 patients (85.45%). Among these, the most common cause was cholesteatomatous chronic otitis media (COM) in 20 patients (36.36%), followed by non-cholesteatomatous COM in 10 patients (18.18%), the presence of a retraction pocket in 5 patients (9.09%), and otosclerosis in 4 patients (7.27%). Less frequent causes included post-trauma with ossicular chain damage in three patients (5.45%) and keratosis obturans in two patients (3.64%). Malformations, both of the external and middle ear, accounted for four patients (7.27%), and the least frequent categories, each with one patient (1.82%), were post-radiotherapy necrosis in a patient with a history of nasopharyngeal carcinoma and iatrogenic external auditory canal stenosis.

On the other hand, among the eight patients with sensorineural hearing loss (14.54%), the most frequently found causes were presbycusis with poor hearing aid outcomes in five patients (9.09%), followed by streptomycin ototoxicity, genetic causes, and labyrinthitis, each with one patient (1.82%).

In this way, the average hearing situation of the patients included in the sample, prior to the placement of the VSB, was as follows: Considering the overall results for all types of hearing loss, we found that the pre-implantation PTA for all the frequency averages was 75.25 ± 20.68 dB, while post-implantation, it reached 33.70 ± 7.71 dB (a gain of 41.56 ± 22.63 dB). The comparison was statistically significant, with a *p*-value < 0.001.

**Figure 4:**
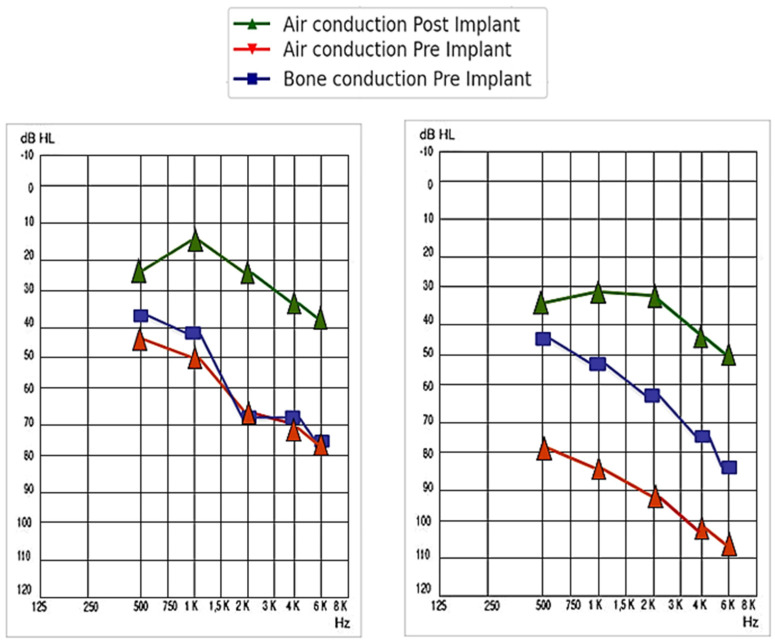
Representation of the audiometric situation pre- and post-VSB placement in patients with sensorineural hearing loss (**left** image) and mixed-conductive hearing loss (**right** image).

For patients with sensorineural hearing loss, the pre-implantation PTA for all the frequency averages was 63.44 ± 13.41 dB, while post-implantation, it reached 32.4 ± 10.12 dB (a gain of 31.04 ± 8.80 dB). On the other hand, for patients with mixed or conductive hearing loss, the pre-implantation PTA for all the frequency averages was 82.59 ± 20.85 dB, while post-implantation, it rose to 39.63 ± 11.01 dB (a gain of 42.96 ± 17.70 dB). The comparison was not statistically significant, with a *p*-value = 0.080. A summary of the audiometric results for each type of hearing loss, by frequency, is shown in Table 2 and represented in Figure 4.

On the other hand, we analyzed the speech discrimination percentage using speech audiometry at 65 dB. For all types of hearing loss, before implantation, the mean score was 11.35 ± 11.33%, while after the placement of the VSB, it was 86.56 ± 18.012%.

For sensorineural hearing loss, before implantation, the result was 22.12 ± 12.33%, while after the placement of the VSB, it was 79.91 ± 9.03%. This implies a gain of 57.79 ± 15.28%. Conversely, in the total patients with conductive or mixed hearing loss, the pre-implantation discrimination percentage was 11.15 ± 13.22%, whereas after the placement of the VSB, it was 80.47 ± 20.73%. This implies a gain of 69.32 ± 24.58%.

### 3.2. Surgical Anchorage and Auditory Results

#### 3.2.1. Coupling in the Round and Oval Window

When studying the gain behavior based on anchorage, we found that out of the total implanted patients, 41 (74.54%) used coupling in the round or oval window, as described in Figure 2. Among these, 24.39% (n = 10) used a coupling system. Of these patients, 39 (95.12%) had conductive or mixed hearing loss, while the remaining two (4.88%) had sensorineural hearing loss.

Considering the overall results for patients with anchorage to the round or oval window, we found that the pre-implantation PTA for all the frequency averages was 79.02 ± 18.91 dB, while post-implantation, it reached 30.23 ± 7.34 dB (a gain of 48.79 ± 21.25 dB). The comparison was statistically significant, with a *p*-value < 0.001.

**Figure 5:**
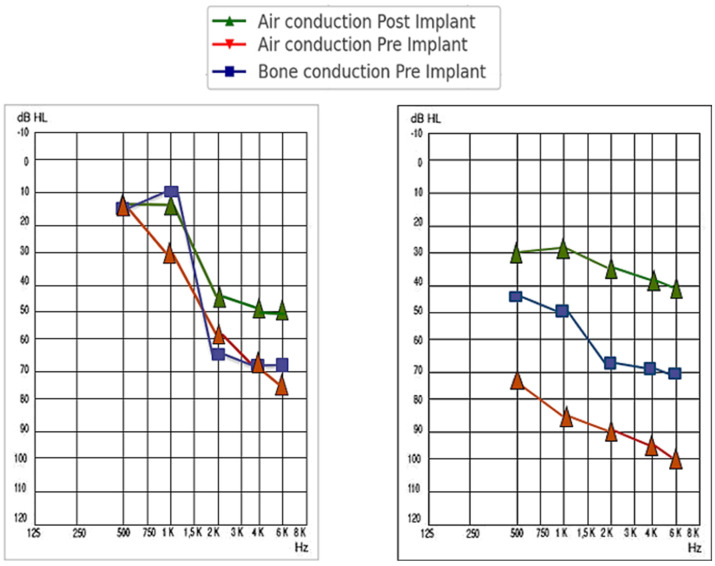
Representation of the audiometric situation pre- and post-VSB placement with round or oval window coupling in patients with sensorineural hearing loss (**left** image) and mixed-conductive hearing loss (**right** image).

For patients with sensorineural hearing loss, the pre-implantation PTA for all the frequency averages was 64.50 ± 17.68 dB, while post-implantation, it reached 25.00 ± 7.07 dB (a gain of 39.50 ± 17.68 dB). On the other hand, for patients with mixed or conductive hearing loss, the pre-implantation PTA for all the frequency averages was 80.23 ± 18.85 dB, while post-implantation, it decreased to 30.67 ± 7.33 dB (a gain of 49.56 ± 20.19 dB). The comparison was not statistically significant, with a *p*-value = 0.449. A summary of the audiometric results for each type of hearing loss in patients who used round or oval window coupling, by frequency, is shown in Table 3 and represented in Figure 5.

When we analyzed the speech discrimination percentage using speech audiometry at 65 dB, we noticed that, for all types of hearing loss, before implantation, the main score was 7.42 ± 10.13%, while after the placement of the VSB, it was 87.73 ± 19.85%. This indicates an improvement of 80.31 ± 17.45%.

For sensorineural hearing loss, before implantation, the result was 25.00 ± 21.21%, while after the placement of the VSB, it was 95.00 ± 7.07%. This implies a gain of 70.00 ± 22.43%. Conversely, in the total number of patients with conductive or mixed hearing loss, the pre-implantation discrimination percentage was 5.96 ± 7.93%, whereas after the placement of the VSB, it was 87.13 ± 20.52%. This implies a gain of 81.17 ± 21.19%.

#### 3.2.2. Ossicular Chain Coupling

In another portion of the sample, we found a total of 14 patients in whom ossicular chain coupling was used. Specifically, in 11 of them (20%), the coupling was carried out at the incus, mainly at the long process in 81.82% (n = 9) via posterior tympanotomy, while in the remaining 18.18% (n = 2), it was performed at the short process via extended atticotomy. These differences are illustrated in Figure 1. In the remaining three patients (21.42%), the FMT was anchored to the stapes. Similarly, the same proportion of patients (21.42%) used a coupler system involving the ossicles. Out of the 14 patients, 8 of them (57.14%) had mixed hearing loss, while the remaining 42.86% (n = 6) had sensorineural hearing loss.

Considering the overall results for the patients with ossicular chain anchorage, we found that the pre-implantation PTA for all the frequency averages was 67.98 ± 14.71 dB, while post-implantation, it reached 35.70 ± 9.13 dB (a gain of 32.28 ± 14.71 dB). The comparison was statistically significant, with a *p*-value < 0.001.

**Figure 6:**
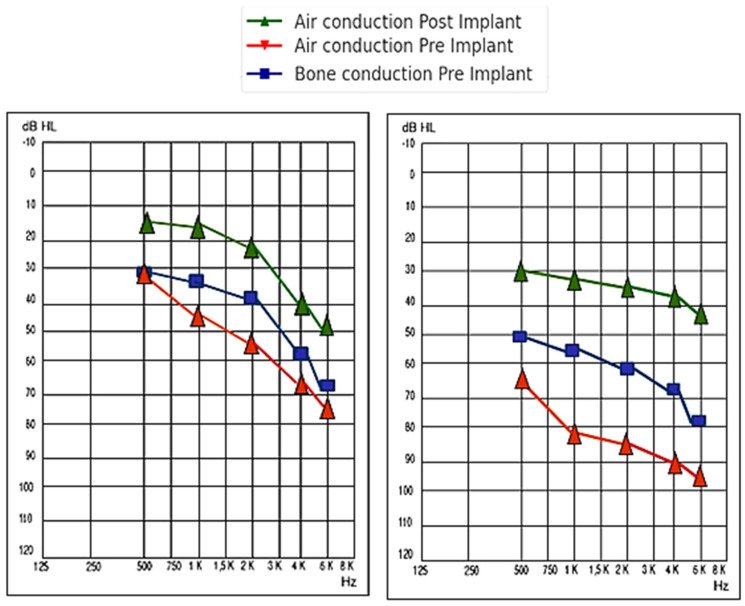
Representation of the audiometric situation pre- and post-VSB placement with incus or stapes coupling in patients with sensorineural hearing loss (**left** image) and mixed-conductive hearing loss (**right** image).

For patients with sensorineural hearing loss, the pre-implantation PTA for all the frequency averages was 62.22 ± 15.90 dB, while post-implantation, it reached 31.33 ± 9.83 dB (a gain of 30.88 ± 15.90 dB). On the other hand, for patients with mixed or conductive hearing loss, the pre-implantation PTA for all the frequency averages was 70.45 ± 14.04 dB, while post-implantation, it decreased to 37.57 ± 8.48 dB (a gain of 32.88 ± 14.04 dB). The comparison was not statistically significant, with a *p*-value of 0.753. A summary of the audiometric results for each type of hearing loss is presented in Figure 6 and Table 4.

When we analyzed the speech discrimination percentage using speech audiometry at 65 dB, we noticed that, for all types of hearing loss, before implantation, the main score with ossicular chain anchorage was 17.85 ± 16.31%, while after the placement of the VSB, it was 84.55 ± 21.24%. This implies an improvement of 66.70 ± 17.45%, and the difference between both moments was statistically significant, with a *p*-value < 0.001.

For sensorineural hearing loss, before implantation, the result was 28.67 ± 21.79%, while after the placement of the VSB, it was 70.17 ± 27.41%. This implies a gain of 41.50 ± 22.43%. Conversely, in the total number of patients with conductive or mixed hearing loss, the pre-implantation discrimination percentage was 13.21 ± 11.37%, whereas after the placement of the VSB, it was 90.71 ± 15.30%. This implies a gain of 77.50 ± 21.19%. This difference was statistically significant, with a *p*-value < 0.001, after conducting *t*-tests.

### 3.3. Improvements in Speech Discrimination at 65 dB

**Figure 7:**
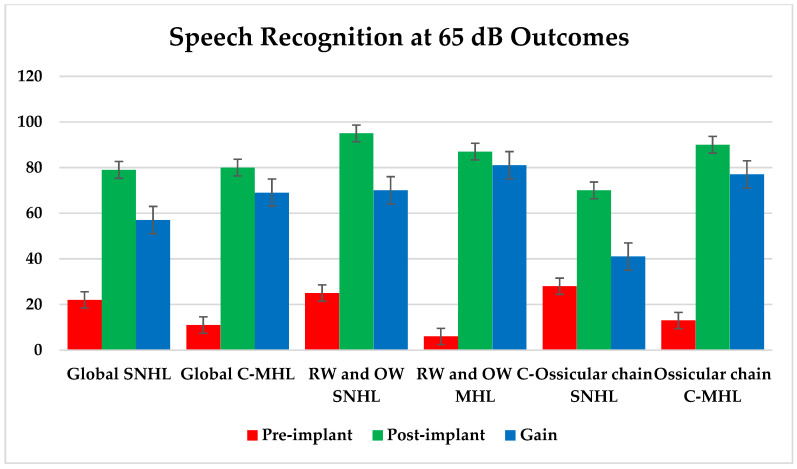
Representation of speech discrimination performance with different couplings and for various types of hearing loss. Error bars represent the 95% confidence interval, providing a visual representation of the variability and reliability of the data.

The evolution in the percentage of discrimination at 65 dB is summarized in Figure 7.

### 3.4. Surgical Technique for Chronic Otitis Media and Auditory Results

Regarding the surgical techniques used to treat complications derived from chronic otitis media (COM) in implanted patients, we found that in 21 cases (70% of patients with chronic otitis media), an open technique was used. This group included 16 cases of canal wall down and 5 cases of attic exposure–antrum exclusion (AE-AE). In the other group, five cases (30%) underwent a closed mastoidectomy technique or canal wall up.

For patients treated with an open technique and implanted with a VSB, the average pre-implantation PTA was 73.55 ± 16.79 dB, which, after the middle ear implant, became a PTA of 41.78 ± 19.02 dB, resulting in an average gain of 43.56 ± 19.01 dB. Regarding speech audiometry information, the average discrimination percentage at 65 dB in patients treated with this surgical technique, before the VSB placement, was 26.81 ± 6.67%. After implantation, it increased to 81.81 ± 7.41%, indicating a gain of 55.00 ± 7.81%.

In contrast, for patients who underwent a closed mastoidectomy surgical technique, an average PTA gain of 56.53 ± 10.57 dB was observed, as the pre-implantation PTA was 90.42 ± 17.53 dB and post-implantation it was 33.89 ± 6.96 dB. Similarly, the gain in terms of the discrimination at 65 dB was 55.11 ± 7.15%, since it was 25.0 ± 6.81% before the VSB implantation and 80.11 ± 6.56% afterwards.

When comparing the PTA gains and speech discrimination according to the surgical technique (open technique vs. canal wall up), we found that comparing the PTA gain between both techniques, the *p*-value was 0.159, indicating that there were no statistically significant differences. Similarly, the gain recorded with speech audiometry was not statistically significant, with a *p*-value of 0.187. A summary of the results according to different surgical techniques is presented in Table 5.

#### Complications and Adverse Effects

Finally, it was observed that a total of nine patients experienced adverse effects derived from the placement of the VSB. Four of them (44.44%) had to undergo reoperation due to cable migration and FMT displacement, with three of these patients having an open mastoidectomy cavity. Another four patients experienced pain in the area of the audioprocessor, which was managed with analgesics, while the remaining patient (11.11%) suffered a skin ulceration due to magnet overpressure, requiring retroauricular plasty for correction.

However, when we compared the risk of experiencing adverse effects between the two surgical techniques, Fisher’s test result gave us a *p*-value of 0.356, indicating that there was no statistically significant relationship.

## 4. Discussion

From a global perspective, the results for this series of patients indicate the effectiveness of the VSB device, even when using different anchoring methods, as an outstanding treatment option in subjects with mixed–conductive and sensorineural hearing loss, with degrees of hearing loss ranging from moderate to severe. Furthermore, the results underscore the role of middle ear active implants as an alternative in conditions where a sufficiently satisfactory gain cannot be achieved with other devices, such as hearing aids, when bone conduction levels fall outside the range for bone-anchored implants, or even in the event of unsatisfactory stapedectomy surgeries in patients with otosclerosis. Similarly, the VSB option may be particularly attractive in cases of chronic otitis media with poor response to tympanoplasty, or in the presence of pathological findings in the external ear that prevent a hearing aid from fitting properly within the external auditory canal, exacerbating poor self-cleaning.

Firstly, the indications for VSB implantation in our cohort reflect the most common etiologies, with cholesteatomatous chronic otitis media being the most prevalent, ac-counting for 36.36% of cases, followed by non-cholesteatomatous chronic otitis media (18.18%) and the presence of a retraction pocket (9.09%). These data are consistent with the literature, where chronic otitis media is also highlighted as one of the main indications for this type of implant [11]. Additionally, other etiologies, such as otosclerosis and presbycusis, are also commonly indicated in both our work and in the literature for the use of the VSB.

Regarding the amplification achieved with the VSB, for all types of hearing loss, a gain of 41.56 ± 22.63 dB was reached. In SNHL, the gain was 31.04 ± 8.80 dB and 42.96 ± 17.70 dB in the case of mixed–conductive hearing loss. These values are almost the same as those reported by Luetje et al., who found an average gain of 31.40 dB at the long-term follow-up for the whole sample [12]. Similarly, other studies have also reported significant auditory gains of a similar magnitude with VSB implantation, particularly at higher frequencies, highlighting the effectiveness of this device in improving hearing in patients with mixed and sensorineural hearing loss [13,14]. In fact, in our study, gains reaching 32.71 ± 18.21 dB at 4000 Hz in SNHL and 49.25 ± 20.26 dB in C-MHL were observed, with this finding being even more pronounced at 6000 Hz, with gains of up to 39.43 ± 9.15 dB in SNHL and 51.16 ± 17.48 dB in C-MHL.

An interesting observation in our study is the notable amplification achieved with the placement of the FMT in the coupling of round and oval windows. This is particularly relevant in terms of average tonal thresholds, both in SNHL (32.00 ± 9.05 dB) and in C-MHL (47.11 ± 3.76 dB). This great efficiency is possibly due to the VSB’s ability to better transmit vibrations to the cochlear fluids through the round window, thereby optimizing the auditory response [15,16]. Additionally, in these cases, there is an inversion of endolymphatic propagation, which goes from the round window to the oval window and not vice versa, as occurs in healthy subjects.

Similarly, in terms of the results provided by speech audiometry, measuring speech intelligibility at 65 dB, it was observed that, especially in patients with C-MHL, the gain was approximately 13% higher (76.64 vs. 63.00%) and was also statistically significant.

To try to understand the reason for this significant superiority when coupling the FMT to a RW or a OW, it could be explained by different phenomena: initially, by the ability to effectively transmit high-frequency vibrations through the round window [17,18,19], thereby improving audibility in these critical frequencies for speech comprehension. Secondly, the importance of greater amplification at high frequencies for consonant discrimination, in turn, has a positive and significant impact on speech intelligibility [20,21]. Subsequently, direct coupling of the FMT to the round window could minimize potential distortions that may occur with other anchoring points, such as the incus, and avoid possible mechanical interferences that can arise with other coupling points. Similarly, as described by Beltrame et al., the use of a coupling system during RW and OW implantation could further improve these results by providing additional stability and ensuring efficient vibration transmission [17]. 

Regarding surgical techniques, we did not find any significant differences in the auditory gain between open and closed techniques. This finding is consistent with other studies that also did not demonstrate any clear differences in auditory outcomes between the two techniques [22]. The choice of technique seems to depend more on the underlying pathology and the surgeon’s preference than on the implant’s efficacy itself [23].

However, in our work, although it did not reach statistical significance, a higher incidence of adverse effects was observed with the open technique. This finding can be attributed to the more invasive nature of this approach, which may increase the risk of postoperative complications, such as infections due to inefficacy in self-cleaning or a larger surface area of previously operated cavities that complicate cable coupling. Previous experiences [24] also indicate that more invasive procedures are associated with a higher rate of complications, reinforcing our findings. As observed in our study, patients undergoing open techniques showed a higher incidence of adverse effects, especially related to cable migration, compared to those who underwent a more conservative technique.

### Study Limitations

Although the sample size is considerably large, with a total of 55 individuals, the heterogeneity in etiologies and demographic characteristics could influence the generalization of the results. Different etiologies may respond differently to VSB implantation, complicating the interpretation of the overall data.

Moreover, being a single-center study, variability in the choice of surgical techniques (open vs. closed) and FMT anchoring sites (incus vs. round window) may introduce an additional source of variation in the auditory results. Although an attempt was made to control these variables in the analysis, the inherent variability in individual surgical skills and preferences could have influenced the outcomes.

To conclude, another limitation is not having been able to conduct quality of life questionnaires for patients to express their subjective impressions and evaluations of the devices in everyday situations. Patient evaluation complements the results of audiometric tests. Despite the existence of questionnaires like the APHAB (Abbreviated Profile of Hearing Aid Benefit), a future challenge could be to consider a modification of this or a new version specifically for this type of implant.

## 5. Conclusions

This study reaffirms the effectiveness of the Vibrant Soundbridge implant in im-proving hearing in patients with mixed, conductive, and sensorineural hearing loss, with significant auditory gain, especially at higher frequencies, particularly through the round window membrane approach, possibly related to better transmission of vibrations to cochlear fluids, optimizing the amplification of critical frequencies for speech intelligibility.

Regarding surgical techniques, no significant differences in auditory gain were observed between open and closed techniques, although the open technique was associated with a high incidence of postoperative complications, requiring reoperation on several occasions.

Finally, the study highlights the need for an individualized approach to VSB implantation, considering the anatomical characteristics and specific auditory needs of each patient, to optimize the outcomes.

## Figures and Tables

**Table 1:** Summary of the demographic data of the patients included in this study.

Demographic Description				
**Age at treatment**	62.58 ± 17.83 (12–87) years			
**Gender**	29 (52.72%) Women		26 (47.28%) Men	
**Follow-up**	11.54 ± 4.77 years (2.89 months–23.16 years)			
**Ear**	26 (47.28%) Left	26 (47.28%) Right		3 (5.45%) Bilateral

**Table 2:** Summary of the general audiometric results in patients with SNHL or C-MHL.

Frequency/dB HL	Sensorineural Hearing Loss (n = 8)			Conductive–Mixed Hearing Loss (n = 47)		
	AC Pre	BC Pre	AC VSB	AC Pre	BC Pre	AC VSB
500 Hz	44.48 ± 19.12	37.20 ± 28.31	26.67 ± 19.44	80.19 ± 26.44	44.07 ± 21.39	36.17 ± 22.41
1000 Hz	51.26 ± 16.88	40.09 ± 16.98	16.02 ± 12.77	85.79 ± 18.32	52.19 ± 18.90	31.91 ± 19.63
2000 Hz	67.77 ± 12.31	68.22 ± 15.38	27.31 ± 22.46	91.82 ± 11.08	62.21 ± 16.32	31.11 ± 15.44
4000 Hz	72.88 ± 18.33	67.85 ± 23.22	36.44 ± 17.36	102.24 ± 23.75	73.95 ± 12.12	47.22 ± 17.89
6000 Hz	77.23 ± 17.14	75.43 ± 19.78	38.22 ± 18.24	107.36 ± 19.11	82.29 ± 21.47	50.06 ± 21.07
Mean	62.72 ± 16.91	57.36 ± 21.49	28.53 ± 18.56	93.48 ± 20.57	62.54 ± 18.45	39.29 ± 19.67

**Table 3:** Summary of audiometric results in patients with SNHL or C-MHML using RW or OW coupling.

Frequency/dB HL	Sensorineural Hearing Loss (n = 2)			Conductive–Mixed Hearing Loss (n = 39)		
	AC Pre	BC Pre	AC VSB	AC Pre	BC Pre	AC VSB
**500 Hz**	15.50 ± 2.50	15.00	15.00	72.64 ± 21.20	43.22 ± 18.33	30.07 ± 8.24
**1000 Hz**	32.25 ± 1.88	10.25 ± 5.21	15 ± 2.50	86.55 ± 18.66	49.19 ± 9.65	28.19 ± 10.57
**2000 Hz**	60.25 ± 4.00	64.75 ± 3.22	45.00 ± 10.00	90.89 ± 14.44	67.24 ± 18.38	36.12 ± 12.24
**4000 Hz**	68.75 ± 3.25	68.50 ± 2.77	49.97 ± 1.52	96.00 ± 19.81	69.05 ± 10.18	38.33 ± 19.54
**6000 Hz**	75.00 ± 2.77	68.50 ± 3.12	50.73 ± 0.76	100.78 ± 19.16	71.30 ± 17.34	41.29 ± 11.89
**Mean**	50.75 ± 25.20	45.40 ± 27.03	35.94 ± 19.34	89.37 ± 9.63	60.06 ± 14.78	34.80 ± 12.50

**Table 4:** Summary of the audiometric results in patients with SNHL or C-MHML using incus or stapes coupling.

Frequency/dB HL	Sensorineural Hearing Loss (n = 6)			Conductive–Mixed Hearing Loss (n = 8)		
	AC Pre	BC Pre	AC VSB	AC Pre	BC Pre	AC VSB
500 Hz	31.65 ± 8.11	30.08 ± 7.65	14.95 ± 11.23	63.52 ± 13.26	50.17 ± 6.96	30.18 ± 4.73
1000 Hz	43.57 ± 13.44	44.29 ± 12.19	16.02 ± 4.76	82.18 ± 7.44	56.37 ± 14.33	32.19 ± 12.54
2000 Hz	55.32 ± 19.22	53.69 ± 3.44	22.11 ± 8.06	84.96 ± 14.44	60.62 ± 10.72	37.26 ± 14.67
4000 Hz	66.82 ± 14.89	67.33 ± 9.21	41.57 ± 7.20	90.03 ± 20.18	67.23 ± 3.89	39.82 ± 3.28
6000 Hz	75.77 ± 11.06	73.85 ± 12.89	49.51 ± 11.04	92.65 ± 7.58	79.66 ± 14.30	43.59 ± 13.29
Mean	54.63 ± 13.34	53.85 ± 9.08	28.83 ± 8.46	82.67 ± 13.45	62.81 ± 10.04	34.84 ± 9.24

**Table 5:** Comparison of the audiometric results obtained with open and closed techniques, both in terms of pure tone audiometry and speech audiometry, with and without the use of an implant.

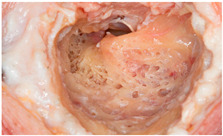	Open Cavity (n = 21)	Canal Wall Up (n = 5)	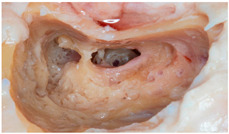
PTA pre-implant	75.33 ± 16.79	90.42 ± 17.53	PTA pre-implant
PTA post-implant	31.77 ± 8.91	33.89 ± 6.96	PTA post-implant
Gain	43.56 ± 19.01	56.53 ± 10.57	Gain
Speech intelligibility pre-implant	26.81 ± 6.67	25.00 ± 6.81	Speech intelligibility pre-implant
Speech intelligibility post-implant	81.81 ± 7.41	80.11 ± 6.56	Speech intelligibility post-implant
Gain	55.00 ± 7.81	55.11 ± 7.15	Gain

## Data Availability

Data pertaining to this study can be shared upon request to the corresponding author.
